# Estimating COVID Risk During a Period of Pandemic Decline

**DOI:** 10.3389/fpubh.2021.744819

**Published:** 2021-12-17

**Authors:** Timothy J. J. Inglis, Benjamin McFadden, Anthony Macali

**Affiliations:** ^1^School of Medicine, University of Western Australia, Crawley, WA, Australia; ^2^Marshall Centre for Infectious Diseases, School of Biomedical Sciences, University of Western Australia, Crawley, WA, Australia; ^3^Department of Microbiology, PathWest Laboratory Medicine WA, Queen Elizabeth II Medical Centre, Nedlands, WA, Australia; ^4^School of Physics, Mathematics and Computing, University of Western Australia, Crawley, WA, Australia; ^5^COVID Live, Caroline Springs, VIC, Australia

**Keywords:** COVID-19, SARS-CoV-2, epidemic risk, health threat assessment, health intelligence, community transmission, delta variant

## Abstract

**Background:** Many parts of the world that succeeded in suppressing epidemic coronavirus spread in 2020 have been caught out by recent changes in the transmission dynamics of SARS-CoV-2. Australia's early success in suppressing COVID-19 resulted in lengthy periods without community transmission. However, a slow vaccine rollout leaves this geographically isolated population vulnerable to leakage of new variants from quarantine, which requires internal travel restrictions, disruptive lockdowns, contact tracing and testing surges.

**Methods:** To assist long term sustainment of limited public health resources, we sought a method of continuous, real-time COVID-19 risk monitoring that could be used to alert non-specialists to the level of epidemic risk on a sub-national scale. After an exploratory data assessment, we selected four COVID-19 metrics used by public health in their periodic threat assessments, applied a business continuity matrix and derived a numeric indicator; the COVID-19 Risk Estimate (CRE), to generate a daily spot CRE, a 3 day net rise and a seven day rolling average. We used open source data updated daily from all Australian states and territories to monitor the CRE for over a year.

**Results:** Upper and lower CRE thresholds were established for the CRE seven day rolling average, corresponding to risk of sustained and potential outbreak propagation, respectively. These CRE thresholds were used in a real-time map of Australian COVID-19 risk estimate distribution by state and territory.

**Conclusions:** The CRE toolkit we developed complements other COVID-19 risk management techniques and provides an early indication of emerging threats to business continuity.

## Introduction

Australia's recent experience with COVID demonstrates how complex disease control becomes during pandemic decline. Australia's early emergence from the first wave of the COVID-19 pandemic gave public health authorities reason to hope for disease elimination, but slow vaccine rollout and the emergence of new SARS-CoV-2 variants have dented that early confidence. The struggle to maintain low COVID prevalence with the limited range of interventions that worked in 2020 has become increasingly challenging. The indicators used to chart epidemic activity during the first pandemic wave were supplemented with other less familiar metrics that communicate subtle changes in the local transmission risk. The national growth factor, originally promoted as a summary of Australia's progress against COVID-19 (1), did not capture differences in COVID-19 epidemiology between Australian states and territories, where pandemic response is delivered. As the first Australian pandemic wave eased we took a strategic look at pandemic response planning (2), and recognised the need for a predictive estimate of COVID risk to guide state-level pandemic responses. We took continuously reported data from each state and territory, and subjected it to multidimensional analysis to achieve a contemporaneous measure of risk. In this account we report our experience generating actionable COVID risk estimates continuously for over a year during the gradual decline of the national COVID epidemic, with interruptions by state-wide and multi-state outbreaks.

## Methods

**Figure 1:**
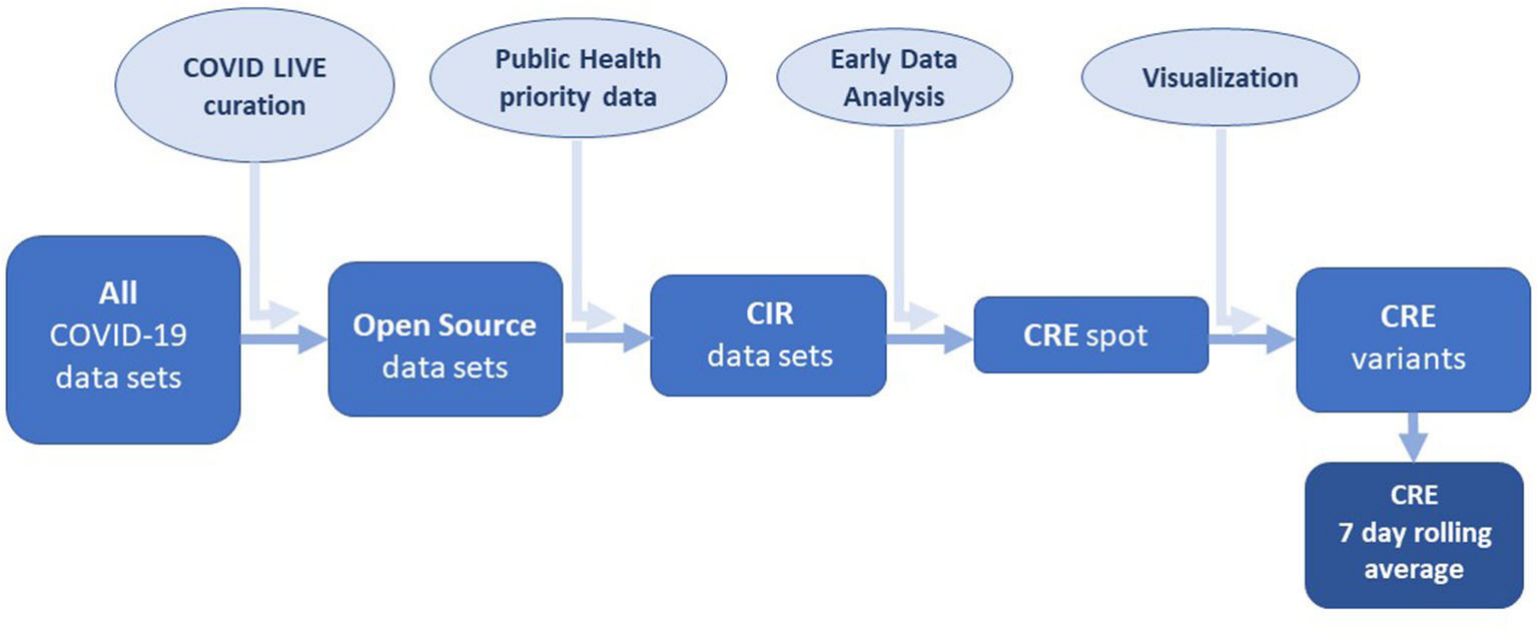
COVID-19 Risk Estimate (CRE) development flow chart: CIR, Critical Information Request.

**Figure 2:**
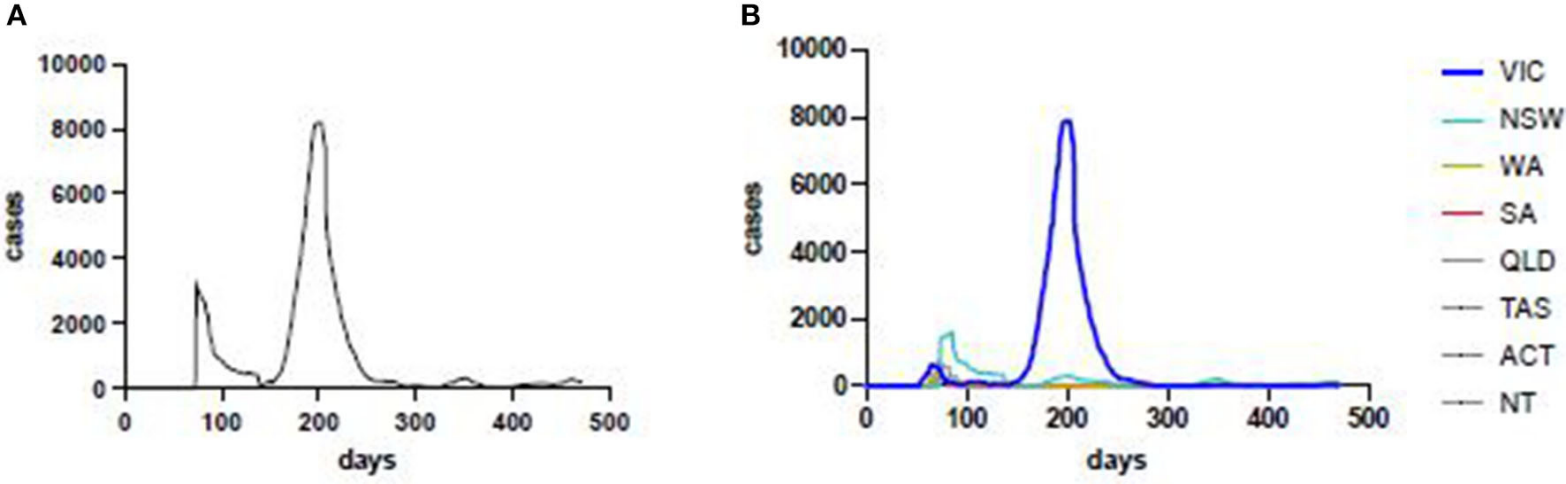
Laboratory-confirmed COVID-19 cases, in Australia (left) and by state and territory (right). VIC, Victoria; NSW, New South Wales; WA, Western Australia; SA, South Australia; QLD, Queensland; TAS, Tasmania; ACT, Australian Capital Territory; NT, Northern Territory.

**Figure 3:**
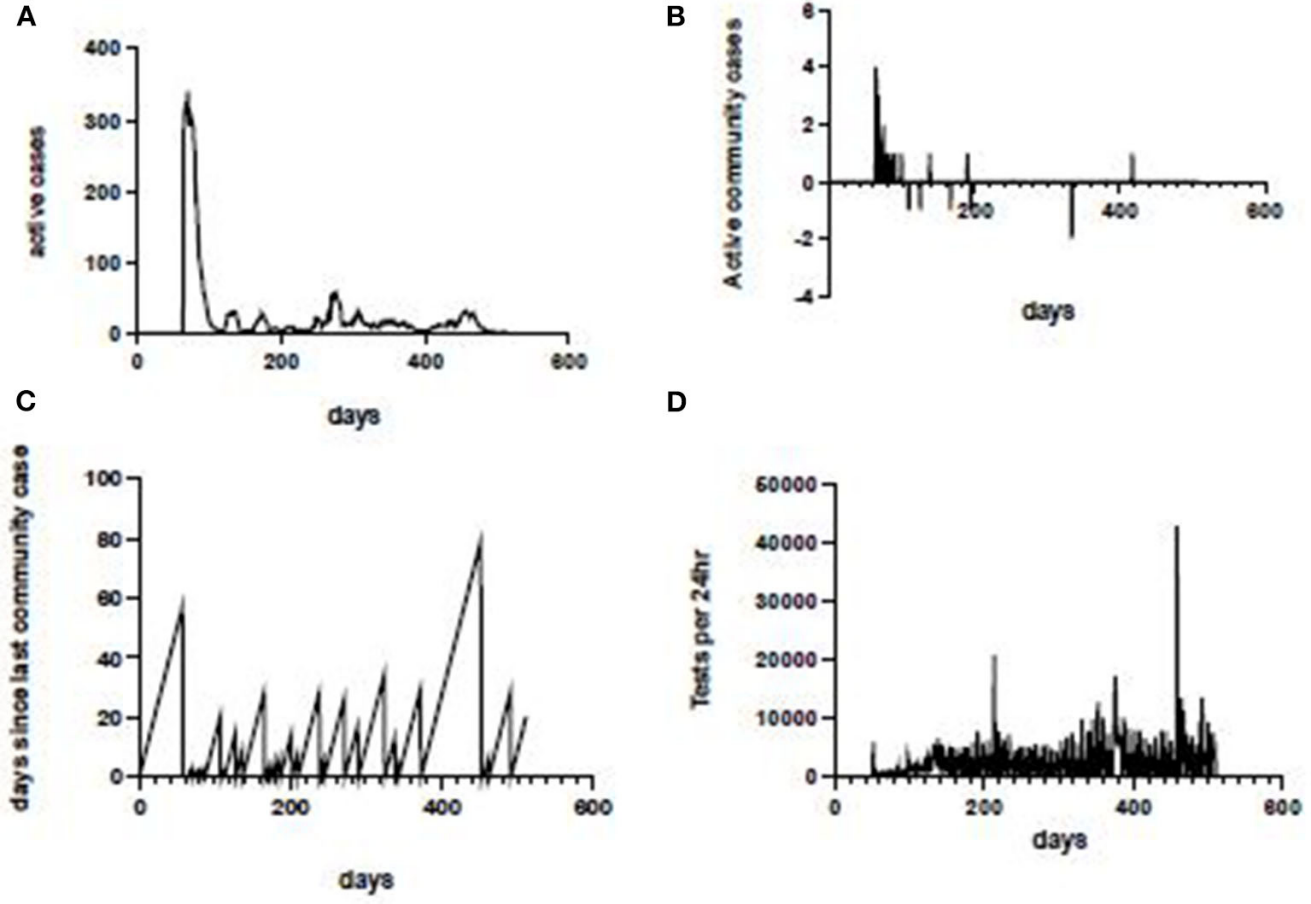
COVID-19 critical information, Western Australia. Laboratory-confirmed COVID-19 cases in Western Australia(WA) (top left); active community cases in WA (top right); interval in days since last community case in WA (bottom left); total RT-PCR tests for SARS-CoV-2 conducted in WA each 24 h (bottom right).

Data source and exploratory data analysis. [The process used to develop the COVID-19 Risk Estimate is shown in a flow diagram (Figure 1)]. Active case data from each Australian state and territory were collated and updated daily on the COVID LIVE website. Conventional epidemic curves were plotted for the entire Australian population and by sub-national jurisdictions (states and territories, Figure 2). Shown on this scale, the active case data from other states and territories appeared trivial by comparison, despite small numbers of community cases being a driver of continued transmission. Exploratory data analysis (EDA) of the principal epidemiological indicators used by public health and other epidemic response agencies, identified three additional critical information requirements: the number of active cases in the community with an unknown source, the number of days since last community transmission and the number of tests performed. These four COVID Critical Information Requirements (CIRs) were then plotted individually against a time axis (Figure 3). These four metrics were reported daily on COVID LIVE since they first became available, but required expert insight to interpret and convert them into a call to public health action.

COVID-19 risk estimate calculation. We incorporated the above metrics into a measurement of COVID risk at state or territory level to monitor trends in COVID risk and, where possible, trigger more detailed data analysis to detect emerging threats. In order to do this we drew on risk assessment methods used in health planning, business risk management and security intelligence to calculate a COVID risk estimate. These metrics were used to populate a daily strength/weakness/opportunity/threat matrix. The allocation of critical variables to this matrix was; days since last community case (strength: DSLCC), number of active cases (weakness: AC), number of tests per day (opportunities: TPD), and the number of active cases in the community with an unknown source (threats: ACUS). To reduce the large discrepancy between axes, these four different metrics were converted to logarithmic values, using the convention that all values <1 were rendered as 1. The calculation was:


$$\begin{array}{l} \text{CR}{\text{E}}_{\text{spot}}=\left[\text{A}{\text{C}}_{\text{log}10}+\text{ACU}{\text{S}}_{\text{log}10}\right]-\left[\text{TP}{\text{D}}_{\text{log}10}=\text{DSLC}{\text{C}}_{\text{log}10}\right] \end{array}$$

**Figure 4:**
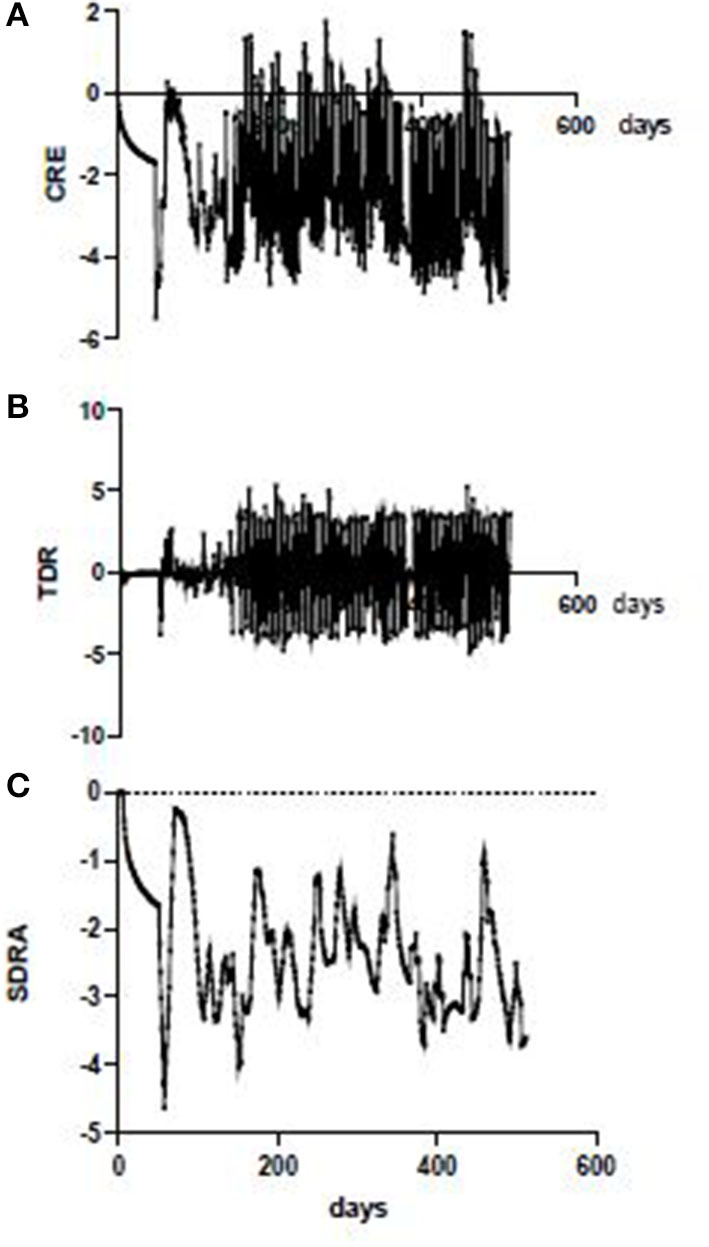
CRE iterations, Western Australia. Daily (spot) CRE in WA showing swinging variations due in part to reporting breaks over weekends (top); nett 3-day rise in CRE (TDR) showing effect of weekend reporting artefact (middle); 7 day rolling average CRE in WA with irregular fluctuation independent of weekend reporting artefact (bottom).

The basic or “spot” COVID Risk Estimate (CRE_spot_) was then used to generate a net 3 day difference (TDR) and a seven-day rolling average to smooth out short-lived artefactual variations due to weekend testing and reporting fluctuations (Figure 4).

## Results

**Figure 5:**
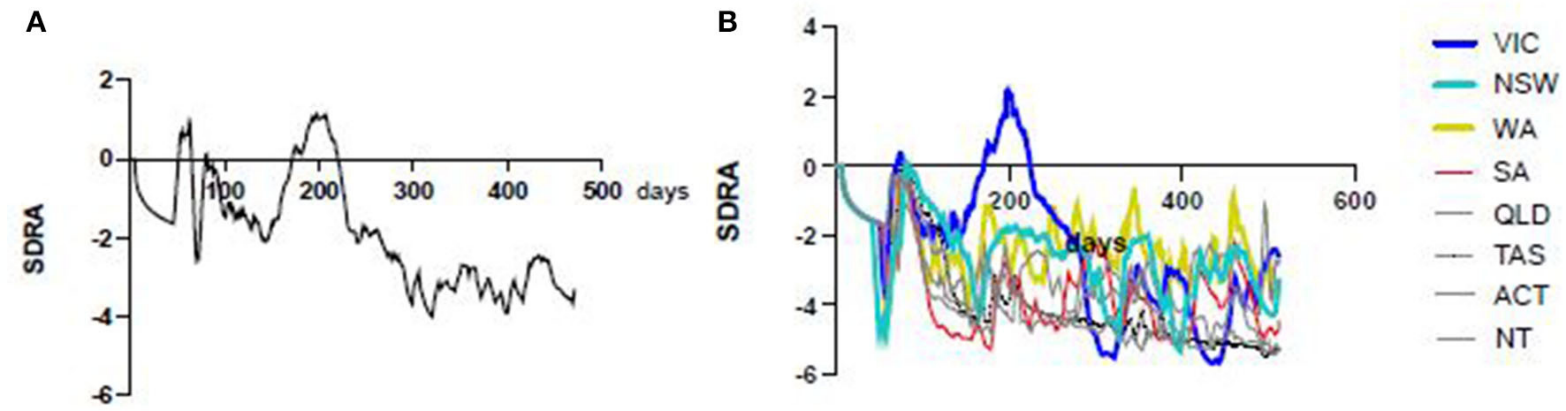
Australian CRE 7 day rolling averages. The all-Australia seven day rolling average CRE shows an overall decline (left) since the start of the pandemic with the exception of a peak around the 200 day mark, largely due to the Victorian second wave (right, blue).

**Figure 6:**
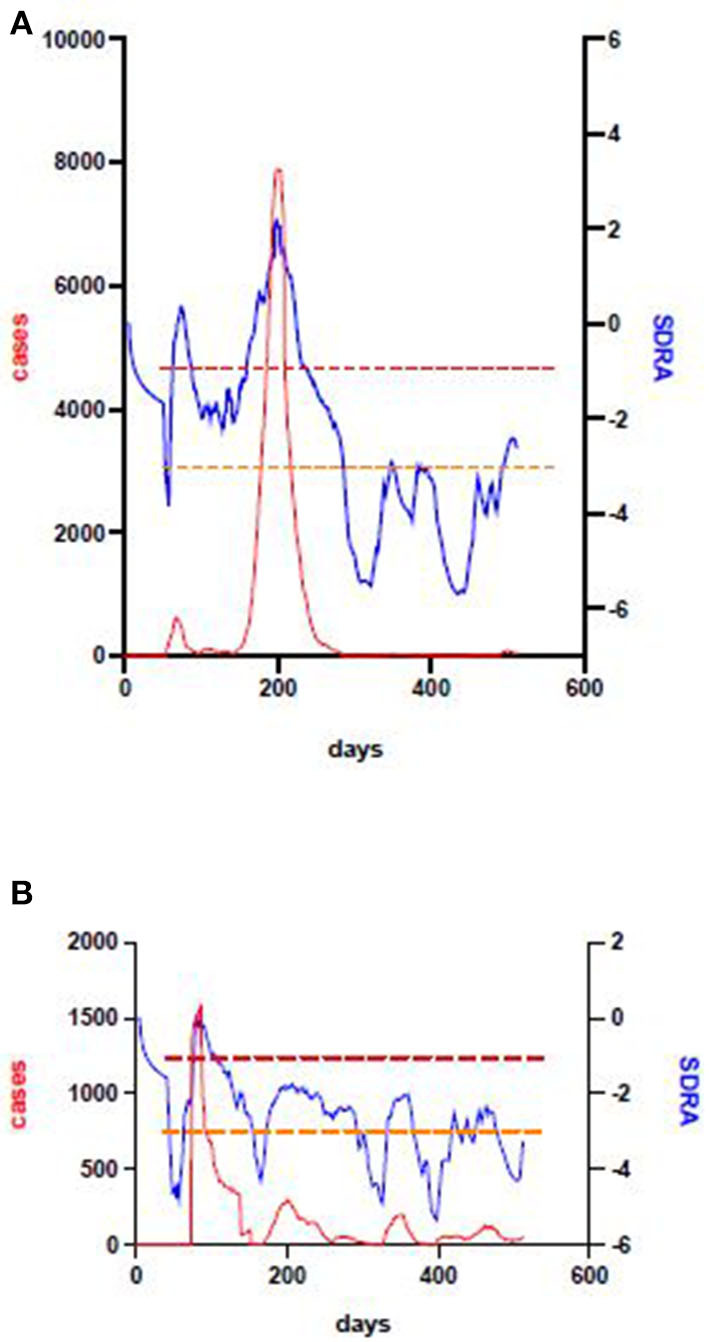
Victoria and New South Wales' second wave. The large outbreak in Victoria during 2020 was preceded by a failure to sustain a declining CRE after the first pandemic wave, a high and fluctuating SDRA baseline (top). This allowed setting lower (orange) and upper (red) thresholds which indicate the point at which outbreaks may propagate, and the higher point above which they will rapidly get out of control, respectively. Application of the same thresholds to the New South Wales SDRA and case data plot show that the same principle applies, even though the total number of cases is much lower.

**Figure 7:**
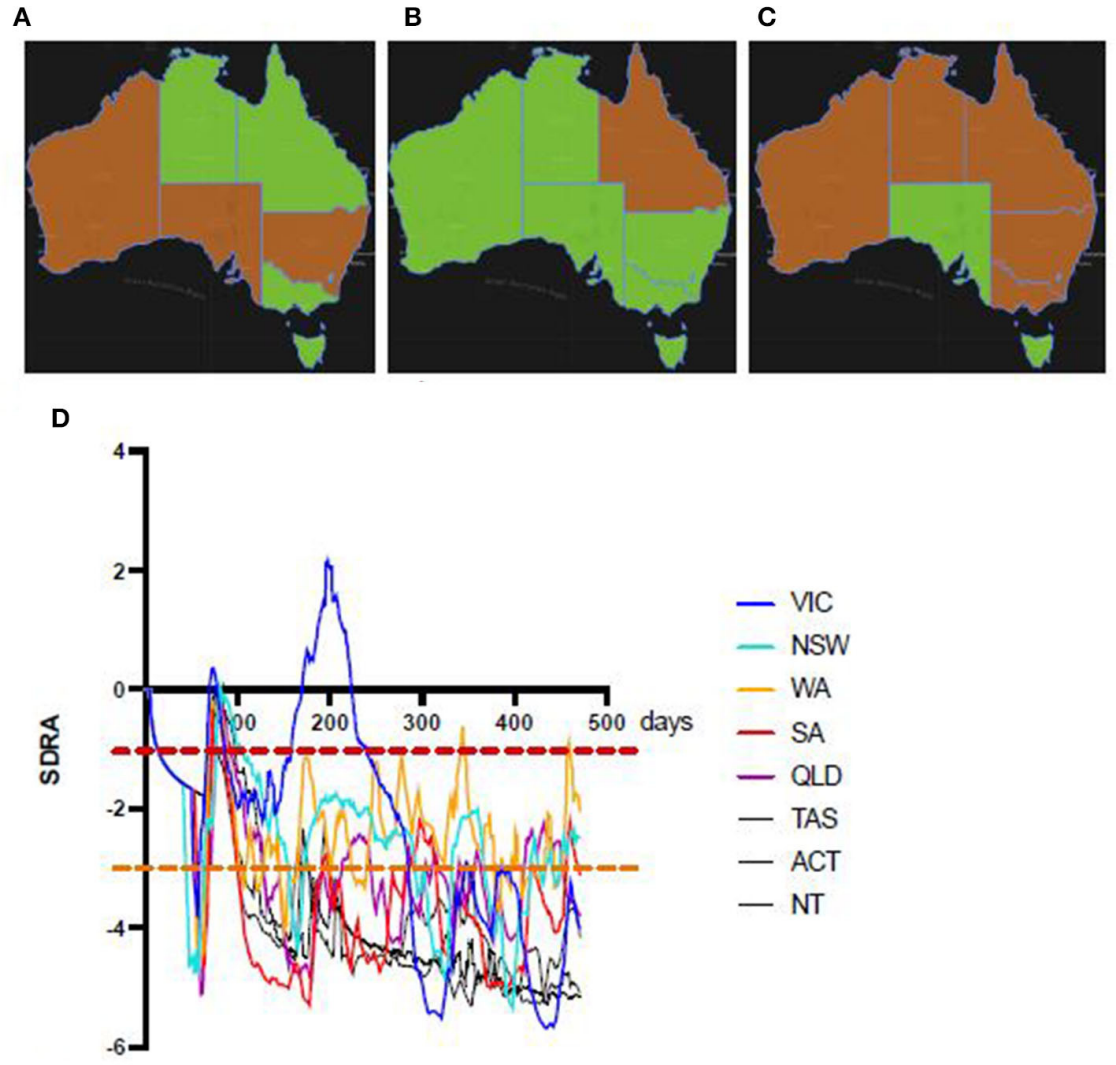
Sequential national snapshots of Australian CRE status. Green (low risk) and orange (medium risk, between lower orange and upper red SDRA thresholds) rating by Australian state and territory on 1st May (left), 1st June (middle) and 1st July (right) show fluctuating CRE status in part due to the recent arrival of the SARS-CoV-2 Delta variant during this period. The corresponding SDRA CRE trend plots by state and territory up to 1st July, 2021 (bottom) show rising CRE baselines in most States and Territories. In New South Wales, Queensland, and Western Australia the CRE (SDRA) had been above the potential propagation threshold for weeks. Noting that the dissemination of the Bondi cluster (NSW) of the Delta variant to other States, the similar pattern to the early stages of the Victorian outbreak suggest a missed opportunity for decisive intervention.

As the first pandemic wave ebbed in every state and territory the seven day rolling average of the COVID risk estimate (SDRA) fell sharply (Figure 5). However, the SDRA decline eased in Victoria sooner than in any other Australian jurisdiction. The Victorian CRE seven day rolling average increased after an oscillating series of small waves before conversion to a sustained, rapid escalation in the weeks prior to the resurgence of COVID-19 in Melbourne. Examination of the active case and active community case metrics during this period in Victoria showed persistent active cases in the community and only short periods since last known community transmission. This allowed the setting of SDRA thresholds for the upper limit of low and of medium risk CRE zones (Figure 6). The medium to high risk transition threshold was placed at the point where the sum total of public health controls was insufficient to contain a sustained rise in active cases. The low to medium transition threshold was set at the point where small clusters of active cases could occur in the community without propagation of an epidemic. The CRE range between these two thresholds represented a zone of medium risk in which the active case number could stabilise, oscillate, continue upwards or fall depending on the computed product of the four CIR inputs. We noted that Western Australia hovered around the low to medium threshold for most of the period despite very little community transmission, underlining the value of sustained public health controls. The national map of upper (red), middle (orange) and lower (green) CRE range (Figure 7) on 1st May, June and July shows a fluctuating CRE status in this series of snapshots, that captures the emergence of the SARS-CoV-2 Delta variant in June and July 2021. The state and territory SDRA plots show a sharp upward trend in affected states and territories (Figure 7B), with the similar starting CRE baselines predicting the need for swift, decisive intervention as seen in the effective short, hard lockdown in Western Australia compared with a more gradual introduction but prolonged control measures in New South Wales.

## Discussion

The Australian CRE data has proved useful as a COVID surveillance tool since the early months of the pandemic. During this period, COVID community transmission has gone into overall decline, with notable exceptions. The lower and upper risk thresholds appear to have held up after the introduction of the national vaccination programme, possibly due to low vaccine uptake and therefore limited impact on transmission. Though public health surveillance, contact tracing, testing and tiered controls have been effective at suppressing COVID throughout Australia, there have been sporadic case clusters linked to leakage from quarantine hotels, fuelled by waning public concern as case numbers fall. Sustaining a high level of vigilance for such a long time requires a concentration of effort, better achieved when a continuously reporting risk estimate method such as our CRE refreshes every state and territory risk estimates daily. For example, the mobile SARS-CoV-2 reverse transcriptase polymerase chain reaction assay method PathWest Laboratory Medicine developed in Western Australia (3) cannot be held in a continual state of high readiness. However, the mobile laboratory can be placed on short-notice, high readiness when the State's CRE crosses the lower threshold, so that deployment can be swift when community transmission demands mobilisation of a surge response.

Emerging variants of concern challenge early diagnosis, effective vaccination and place a heavy burden on scarce hospital resources such as critical care (4). Computational modelling methods have been used for health resource allocation in high prevalence settings where the hospital system in threated with overload (5, 6). This data-driven approach is attractive both for its generation of an evidence base for public health decision-making, and its visualisation tools. Although these big data methods have been applied at a subnational level they are driven by the needs of high COVID prevalence settings. In Australia, the generally low prevalence and pressing need to mount an effective public health response early mean that case numbers are low, while COVID susceptibility and complacency are high. Recently published machine learning methods show promise for short term forecasting of cumulative case load, but only the high prevalence country data was analysed (7). In the present study, access to consistent, open source data stratified at state and territory level prevented more granular analysis e.g., at local government area or postcode level.

The rate of CRE decline eased in Victoria and changed to a wavering baseline pattern before public recognition of the second wave. The extensive disease control interventions required by the Victorian Health Department took many weeks to bring the epidemic under control (8). The subsequent increase in days since last community transmission, increased tests, and reduced active cases, appear as a plummeting CRE. In those states and territories at or below the low to medium CRE threshold, successful disease control has been possible with short-lived lockdown periods, contact tracing and increased testing. Noting this, we were able to use the CRE status of Western Australia to predict the success of a recent snap lockdown (in July, 2021), despite the vulnerability of a state population that previously experienced a full year without community transmission. The propagation of a Delta variant outbreak from Bondi in New South Wales to other states and territories occurred against a CRE repeatedly above the lower (potential propagation) threshold and rapidly spread to other jurisdictions with similar CRE. The New South Wales CRE trend in the preceding weeks followed a similar pattern to Victoria in the lead up to their large 2020 outbreak, as public health authorities stepped up control measures, against an expanding COVID outbreak. The contrast between New South Wales' measured escalation of public health restrictions, and other affected states that quickly introduced lockdowns, appears to be a missed opportunity to deal with this outbreak at source. However, the CRE is not a sophisticated mathematic modelling method. Its principal use is to provide triggers and warnings that prompt a closer look at the available data, so that opportunities for effective early intervention are not missed. For this reason, we believe the CRE is best seen as a form of contemporaneous or real-time operational health intelligence whose immediacy and accessibility help non-expert users to avoid complacency, miss emergency planning opportunities and improve targeted preparedness.

The slow uptake of COVID vaccines in Australia adds further complexity to the epidemiology of pandemic decline in major conurbations and regional centres. A high level of vaccine hesitancy was not helped by promotion campaigns that were geographically disconnected from pandemic response delivery and slowly reactive to major surges. Mobilising public support for increased restrictions was and hampered by political considerations. There is an argument for presenting the seven day rolling CRE average with the upper and lower action thresholds, as a publicly accessible risk rating tool, and aligning the CRE thresholds with the Australian government's hierarchy of controls (eliminate risks; substitute, isolate or reduce the hazard through engineering controls; reduce exposure through administrative controls; use personal protective equipment) (9).

As the world exploits the current range of vaccines to accelerate pandemic decline, we are faced with complex and ambiguous COVID-19 epidemiology. New variants of concern can be blamed for some of this, and may dictate future modification of the CRE toolkit when consistent data reporting is achieved. Experience in low COVID-prevalence countries, such as Australia, provides useful insight into how to monitor, assess and manage residual COVID risk. The Australian COVID Risk Estimate method we developed for local use has the potential to generate timely risk estimate data outside Australia. The CRE seven day rolling average is particularly well suited to public communication of risk, and the net 3 day differential may prove a useful adjunct when mobilising public health emergency responses.

## Data Availability Statement

The datasets presented in this study can be found in online repositories. The names of the repository/repositories and accession number(s) can be found below: https://covidlive.com.au/, https://github.com/benjaminmcf/Australia-CRE.

## Author Contributions

TI generated and developed the original concept, conducted the exploratory data analysis, devised the risk estimate method, ran the trial series of analyses on open source jurisdictional data and engaged stakeholders to set decision thresholds. BM converted the manual calculation method into an automated real-time risk estimate monitor and with TI refined decision thresholds and graphic outputs. AM sourced, presented and maintained the COVID data in an open source repository and provided data extracts to TI and BM throughout the development phase. All authors contributed to the article and approved the submitted version.

## Funding

TI and BM were supported by the Health Department of Western Australia through a translational health research grant to develop COVID countermeasures (FHRIFGCOVID19).

## Conflict of Interest

The authors declare that the research was conducted in the absence of any commercial or financial relationships that could be construed as a potential conflict of interest.

## Publisher's Note

All claims expressed in this article are solely those of the authors and do not necessarily represent those of their affiliated organizations, or those of the publisher, the editors and the reviewers. Any product that may be evaluated in this article, or claim that may be made by its manufacturer, is not guaranteed or endorsed by the publisher.
